# Association of Human Leukocyte Antigen Class II with Susceptibility to Primary Biliary Cirrhosis: A Systematic Review and Meta-Analysis

**DOI:** 10.1371/journal.pone.0079580

**Published:** 2013-11-12

**Authors:** Baodong Qin, Jiaqi Wang, Jia Chen, Yan Liang, Zaixing Yang, Renqian Zhong

**Affiliations:** 1 Department of Laboratory Diagnostics, Changzheng Hospital, Second Military Medical University, Shanghai, China; 2 Department of Stomatology, Changzheng Hospital, Second Military Medical University, Shanghai, China; Emory University School of Medicine, United States of America

## Abstract

**Purpose:**

Several previous studies suggested that *HLA-Class* *II* may be associated with susceptibility to primary biliary cirrhosis (PBC), but data from individual studies remain controversial. Therefore, a systematic review and meta-analysis is needed to comprehensively evaluate the association between *HLA-Class* *II* and PBC risk.

**Methods:**

All published reports of an association between *HLA* class *II* and PBC risk were searched in PubMed, EMBASE (updated to 22 May 2012). ORs with 95% confidence intervals (CIs) were extracted from each included study and the meta-analysis was performed using the fixed- or random-effects model.

**Results:**

A total of 3,732 PBC patients and 11,031 controls from 34 studies were included in the meta-analysis. An assessment of study quality revealed that the majority of studies included (18 studies) were of high quality. The serological group DR8 was found to be a risk factor for PBC (OR = 2.82, 95%CI: 1.84–4.30). At the allelic level, HLA-DR*08 and HLA-DR*0801 were identified as risk factors for PBC (OR = 2.30, 95%CI: 1.76-3.00; OR = 3.23, 95%CI: 2.22–4.70, respectively), whereas HLA-DR*11 and HLA-DR*13 were potent protective factors (OR = 0.31, 95%CI: 0.27-0.38; OR = 0.62, 95%CI: 0.48-0.81, respectively). *HLA-DQB1* and HLA-DQB1*0402 conferred a predisposition to PBC development (OR = 3.47, 95%CI: 2.35–5.13), whereas HLA-DQB1*0604 was protective against PBC (OR = 0.3, 95%CI: 0.18–0.58). No *HLA-DPB1* allele was observed to be associated with PBC susceptibility (*P* > 0.05).

**Conclusions:**

The present study revealed that *HLA-Class* *II* components are closely associated with the development of PBC.

## Introduction

Primary biliary cirrhosis (PBC) is a chronic immune-mediated liver disease characterized by progressive intrahepatic bile-duct destruction, leading to cirrhosis and eventual liver failure [1]. Although the etiology of PBC is unclear, it has been suggested that both genetic and environmental factors may initiate and promote the disease [2]. Specifically, the high concordance for PBC in monozygotic twins, family clustering, and female predominance suggest that genetic factors may play an important role in the development of PBC [3-5]. 

Human leukocyte antigens (HLA) are encoded by genes within the major histocompatibility complex (MHC), located on chromosome 6p21. Genes located in the centromeric class II region of the *HLA* encode polymorphic *HLA-DR*, *HLA-DQ*, and *HLA-DP*, which are expressed on antigen-presenting cells and can participate in antigen processing. Although accurate mechanisms to explain how the *HLA* class *II* gene induces autoimmunity are unclear, several *HLA class II* alleles have been identified to confer genetic risk for or protection against many autoimmune diseases [6], and such associations between certain *HLA class II* alleles and the development of PBC have been extensively investigated.

The most commonly detected *HLA* genes involved in susceptibility to PBC are the Class II DRB1*08 allele family, especially DRB1*0801 and DRB*0803, which were implicated as risk factors for PBC in some studies [7-9]. However, data to suggest an association between DRB1*08 and PBC risk are controversial at this time. In addition to PBC risk conferred by *HLA Class II* alleles, several studies suggest that some alleles could be protective against PBC such as DRB1*11 and DRB1*13 [10,11]. These results support the concept that *HLA Class II* alleles may have diverse roles in PBC pathogenesis. 

In addition to *HLA* alleles, some haplotypes, such as DRB1*0803-DQA1*0103-DQB1*0601 and DRB1*0801-DQA1*0401-DQB1*402, were also found to be associated with PBC risk in studies of patients with diverse ethnic origins [8-10]. Further evidence from recent genome-wide association studies (GWAS) also revealed a strong association between polymorphisms in the *HLA II* region and PBC. In GWASs, the *HLA-DRB1* and *HLA-DQB1* locus were reported to be significantly associated with susceptibility to PBC [12,13]. To better understand GWAS results regarding roles of *HLA* class *II* in PBC, quantitative analyses are needed to pool data and estimate associations. 

Data supporting a protective role for *HLA Class II* alleles is of interest to investigators researching molecular mechanisms of PBC disease development [14]. Although many studies have focused on the involvement of *HLA* genes in PBC, results from these investigations were inconsistent or inconclusive due to small sample sizes. In the view of existing heterogeneity among these studies, we conducted a meta-analysis to comprehensively assess the association between *HLA Class II* and PBC risk. 

## Methods

### Search strategy

A literature search of electronic databases including PubMed, EMBASE, (updated to 22 May 2013) was conducted independently by two investigators (BDQ and JC). The search included only published studies on association between *HLA-Class II* (*HLA-DRB1*, *HLA-DQB1*, and *HLA-DPB1*) and susceptibility to PBC. The search strategies including Mesh terms and keywords were as follows: “primary biliary cirrhosis”, ”human leukocyte antigens”, “major histocompatibility complex”, and “HLA-DR/DQ/DP antigens”. No limit was placed on publication language or geographic area.

### Study selection

Studies included in the meta-analysis fulfilled selection criteria as follows: a) the study should be a case-control design; b) PBC patients should be diagnosed according to internationally accepted criteria; c) the study should contain sufficient published data for the evaluation of an odds ratio (OR) with a 95% confidence interval (95%CI); and d) the study should have been published in journals as full articles. Studies which had one of the following exclusion were discarded: a) the study was based on family or sibling pairs; b) the study had repeated reports on the same population or subpopulation; c) the study had no control group; d) there was insufficient published data for extraction; and e) the paper was a review or abstract.

### Data extraction

All included studies were retrieved and the data were extracted independently in duplicate using a standard protocol by two authors (BDQ and JC). Study characteristics were extracted, including the first author, year of publication, country in which the study was launched, ethnicity of the population sample, *HLA* genotyping technique, sample size, and total number of cases and controls (Table **1**). In the literature review, data were chiefly calculated using two methods. One method compared the difference in the number of individuals who carried *HLA-DRB1*, *HLA-DQB1*, *HLA-DPB1* alleles, comparing PBC patients and controls (***n_a_/N,n*_*a*_** is number of individuals who carried the alleles, ***N*** is the total number of PBC patients or controls). The second method used differences in the frequency of the alleles between PBC patients and controls (***n_b_/2N*, *n*_*b*_** is the number of some alleles and ***N*** is the total number of PBC patients or controls). 

**Table 1:** Characteristics of 34 publications included in the meta-analysis of *HLA* class *II* and PBC susceptibility.

Author	Year	Country	Ethnicity	Sample size	Number of PBC patient/control	Specific technique
Miyamori H, et al.	1983	Japan	Asian	72	22/50	microlymphocytotoxicity
Bassendine MF, et al.	1985	UK	Caucasian	275	75/200	microlymphocytotoxicity
Johnston DE, et al.	1987	UK	Caucasian	200	71/129	microlymphocytotoxicity
Briggs DC, et al.	1987	UK	Caucasian	403	96/307	microlymphocytotoxicity
Gores GJ, et al.	1987	USA	Caucasian	285	114/171	microlymphocytotoxicity
Prochazka EJ, et al.	1990	USA	Caucasian	1,581	35/1,546	microlymphocytotoxicity
Manns MP, et al.	1991	Germany	Caucasian	201	25/176	microlymphocytotoxicity
Underhill J, et al.	1992	UK	Caucasian	321	159/162	PCR/RFLP
Morling N, et al.	1992	Denmark	Caucasian	1,227	23/1,204	microlymphocytotoxicity
Gregory WL, et al.	1993	UK	Caucasian	493	130/363	RFLP
Begovich, et al.	1993	USA	Caucasian	291	51/240	PCR-SSO
Seki, et al.	1993	Japan	Asian	191	41/150	microlymphocytotoxicity/PCR
Onishi S, et al.	1994	Japan	Asian	246	31/215	PCR-SSO
Zhang L, et al.	1994	UK	Caucasian	83	40/43	PCR/RFLP
Mehal WZ, et al.	1994	USA	Caucasian	125	64/61	RFLP
Underhill JA, et al.	1995	UK	Caucasian	185	82/103	PCR-SSO
Mella JG, et al.	1995	Germany	Caucasian	79	32/47	PCR-SSO
Akimoto S, et al.	1999	Japan	Asian	228	13/215	NA
Donaldson P, et al.	2001	UK	Caucasian	266	164/102	PCR-SSO
Stone J, et al.	2002	USA	Caucasian	370	154/216	PCR-SSP
Wassmuth R, et al.	2002	Germany	Caucasian	257	99/158	PCR-SSO
Invernizzi P, et al.	2003	Italy	Caucasian	670	112/558	PCR-SSO
Bittencourt PL, et al.	2003	Brazil	Caucasoid/African American/Amerindian	144	61/83	PCR-SSP
Jiang XH, et al.	2004	China	Asian	95	52/43	PCR-SSP
Mullarkey ME, et al.	2005	USA	Caucasian	453	72/381	PCR-SSO
Chen C, et al.	2005	China	Asian	132	72/60	PCR-SSP
Liu HY, et al.	2006	China	Asian	496	65/431	PCR-SSP
Donaldson PT, et al.	2006	UK/Italy	Caucasian	823	492/331	PCR-SSO
Zhao J, et al.	2006	China	Asian	107	40/67	PCR-SSP
Invernizzi P, et al.	2008	Italy	Caucasian	2,656	664/1,992	PCR-SSO
Vázquez-Elizondo G, et al.	2009	Mexico	Mexican/Spanish	390	9/381	PCR-SSP
Nakamura M, et al.	2010	Japan	Asian	592	334/258	PCR-SBT
Chong VH, et al.	2010	Brunei Darussalam	Asian	74	9/65	PCR-SSO
Umemura T, et al.	2012	Japan	Asian	752	229/523	PCR-SSO
Total	/	/	/	14763	3732/11031	/

Using these methods, data were pooled and a meta-analysis was performed. Any disagreement in the data was resolved by discussion and consensus (The data would be extracted independently by other authors using the same standard protocol). 

### Assessment of study quality

Due to the lack of standardized quality criteria for meta-analyses of single nucleotide polymorphism studies, we chose the Newcastle-Ottawa scale (NOS) to assess the quality of these non-random studies. According to the NOS, the criteria for evaluation include selection of cases and controls, comparability of cases and controls, and ascertainment of exposure [15]. The scoring system provided a summary numeric score of quality that ranged from 0 star to 9 stars. The studies were graded into the three categories: high (>=7 stars), medium (4-6 stars) and low (<=3 stars) quality [16].

### Statistical analysis

The final effect ORs and 95%CIs were calculated in a random-effect or fixed-effect model to evaluate the strength of the association between HLA Class II and PBC risk. Heterogeneity of effects among studies was estimated using the means of χ^2^-based Q test and I^2^ test. For the Q test, a *P* value less than 0.1 was considered to be representative of significant heterogeneity, and an I^2^ statistic represented the percentage of total variation contributed by a between-study variation that ranged from 0 to 100% [17]. If no significant heterogeneity was observed, a fixed-effect model was used to pool the data. Otherwise, a random-effect model was used and meta-regression analysis was used to identify the potential source of heterogeneity if sufficient studies were included. Funnel plots, Egger’s test, and Begg’s test were used to determine publication bias. A sensitivity analysis was performed to test data stability, and a cumulative meta-analysis was used to determine whether the strength of a relationship was stable through the repeated performance of meta-analysis whenever a new study became available for inclusion. The number or frequency of *HLA Class II* was zero in some included studies, so these zero total event studies were included to provide the most generalizable estimated ORs [18]. Studies with no events in both groups were excluded. All analyses were conducted in STATA 11.0 software (Stata Corp, College Station, TX). *P* values less than 0.05 were considered significant.

## Results

### Studies included in the meta-analysis

**Figure 1:**
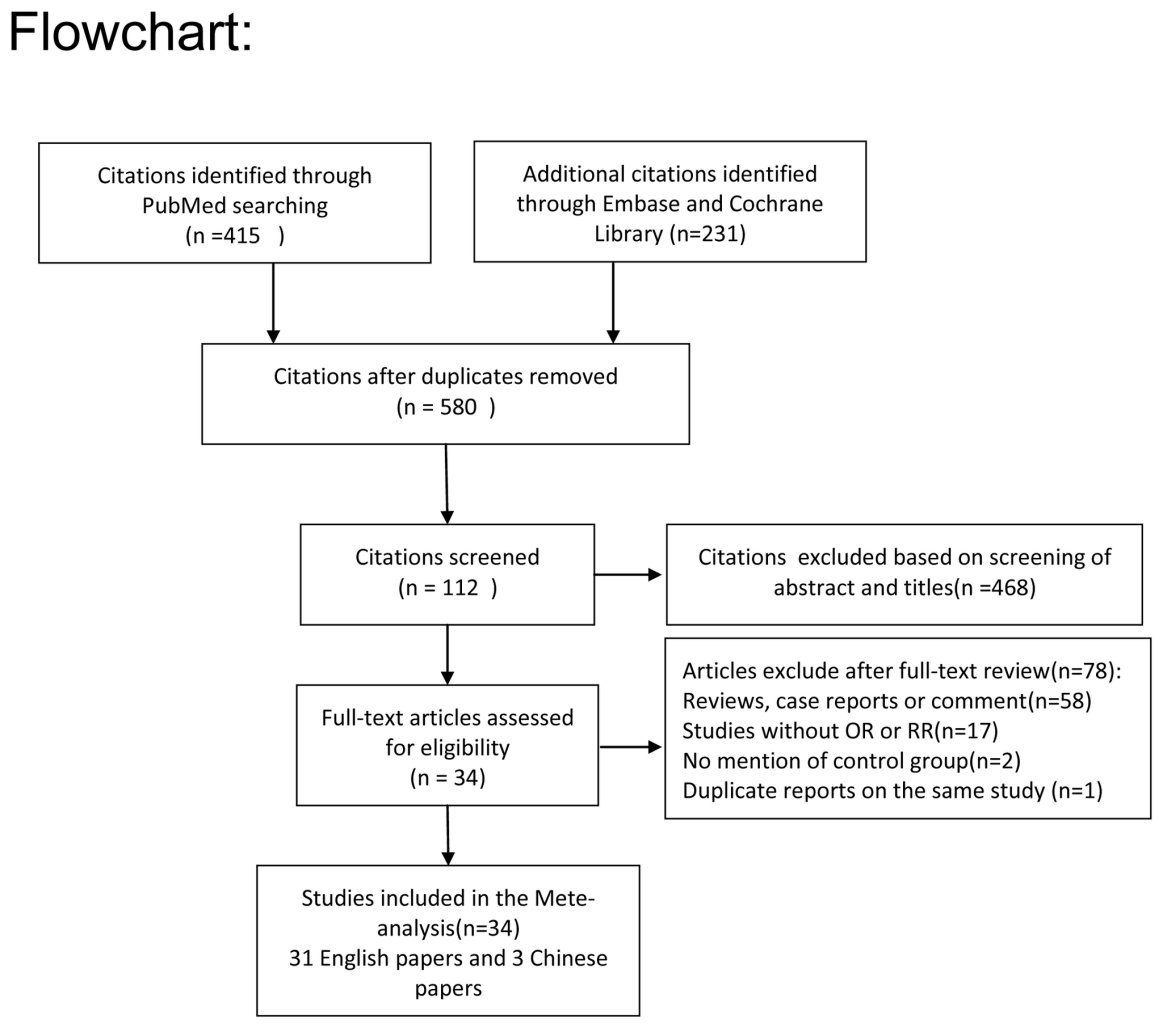
Flowchart of the present meta-analysis.

From the initial published work search, a total of 580 non-overlapping articles were identified and screened from the previously described electronic databases. A total of 468 articles were excluded based on screening of abstracts or titles. After retrieving the full-text articles, 78 articles were excluded based on the exclusion criteria, leaving 34 relevant studies for the meta-analysis [7-11,19-47]. (Figure **1**)

### Characteristics of included studies

The thirty-four studies were published between 1983 and 2012 and were comprised of 31 English-language papers and 3 Chinese-language papers, with data for 3,732 PBC patients and 11,031 controls. All 34 studies fulfilled the inclusion criteria and an OR with a 95%CI could be obtained from each study. Study information and characteristics are depicted in Table **1**. 

### Assessment of study quality

According to the scoring system given, the quality of each study was assessed and 7 studies scored 8 stars, 11 scored 7 stars, 7 scored 6 stars, 7 scored 5 stars and 2 scored 4 stars. (Table **S1**).

### Serological HLA-DR and PBC

A total of 13 studies contained data on serological HLA-DR from 5,400 subjects (788 cases of PBC and 4,612 controls). The meta-analyses for associations between each HLA-DR and PBC susceptibility revealed that only 6 of 16 associations were statistically significant. The serological groups HLA-DR2, DR5, DR12, DR16, and DR52a were potent protective factors against PBC, whereas DR8 was found to be a risk factor for PBC. The pooled ORs and 95%CIs were 2.82 (1.84-4.30), 0.70 (0.53-0.91), 0.55 (0.39-0.78), 0.40 (0.19-0.86), 0.21 (0.05-0.97), and 0.60 (0.38-0.95), respectively, for DR8, DR2, DR5, DR12, DR16, and DR52a. (Figure **S1**)

**Figure 2:**
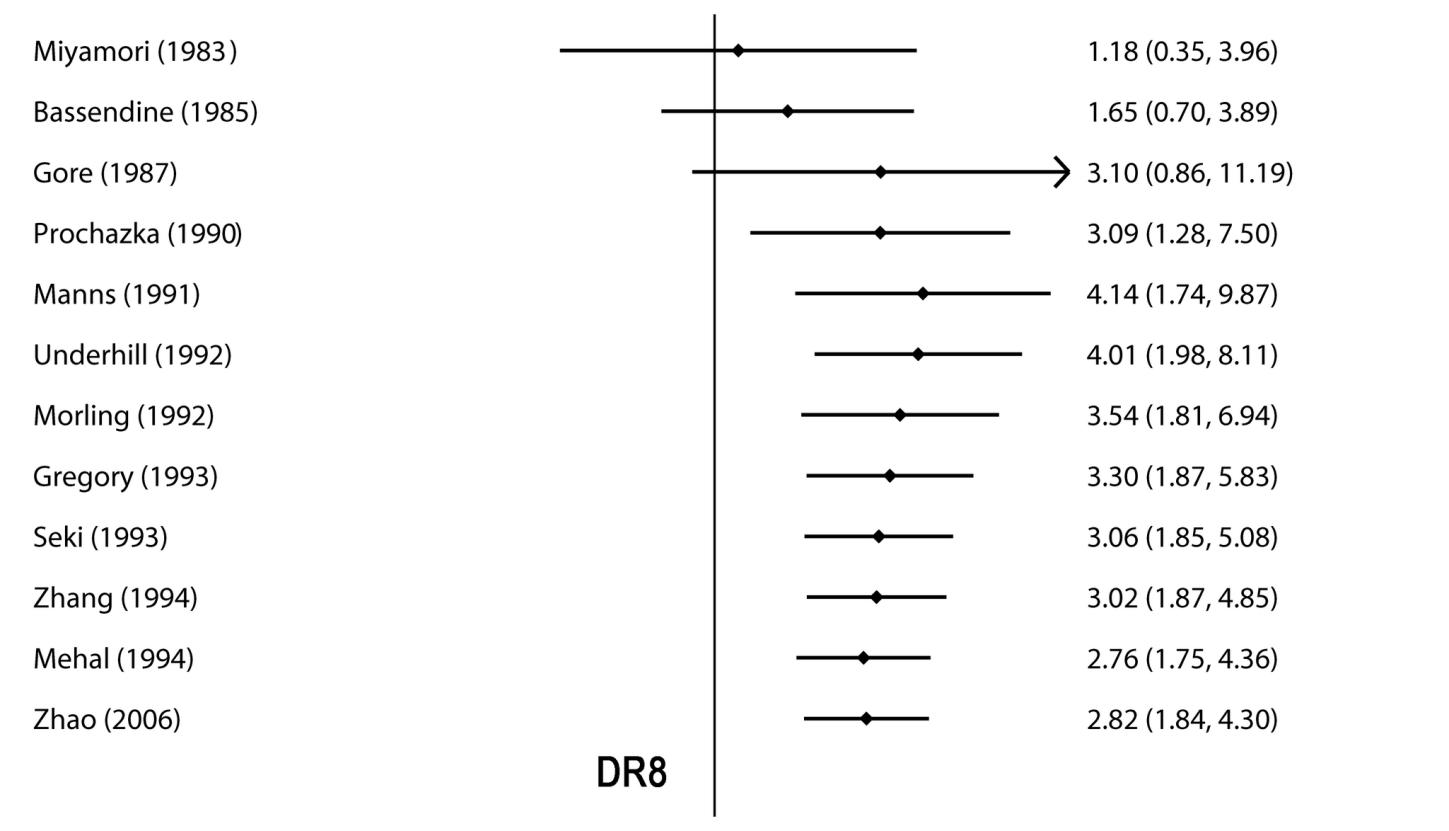
Cumulative meta-analysis of 12 studies of *HLA-DR8* and susceptibility to PBC by the Mantel-Haenszel method with the random-effect model.

There was no heterogeneity among the studies with respect to the association between DR2, DR5, DR12, DR16, and DR52a and the risk of PBC, but a high degree of heterogeneity was found to exist between DR8 and PBC risk (I^2^= 54.8%, *P* = 0.011). A subgroup meta-analysis by ethnicity indicated that a significant association was discovered in both Caucasian and Asian groups, but the Caucasian group was significantly heterogeneous. Subsequently, the cumulative meta-analysis revealed that the estimated OR and 95%CI were stable in order of year of publication and the convergence of evidence across numerous studies confirmed the strength of HLA-DR8 as a risk factor for PBC. A statistically significant association was detected as far back as 1990 [24] (Figure **2**).

### *HLA-DQB1* alleles and PBC

A total of 14 meta-analyses of studies of *HLA-DQB1* alleles and PBC susceptibility were conducted. *HLA-DQB1* (*02, *04, *0401, *0402 and *0601) were found to be risk factors for PBC, and the pooled ORs and 95%CI were 1.40 (1.00–1.97), 2.24 (1.46–3.46), 1.41 (1.07–1.85), 3.47 (2.35–5.13), and 1.99 (1.57–2.53), respectively (Figure **S2**). In contrast, *HLA-DQB1* (*0301, *06, *0602 and *0604) were protective factors, and the pooled ORs and 95%CIs were 0.62 (0.48–0.79) and 0.48 (0.35–0.68), 0.58 (0.39–0.85), 0.61 (0.42–0.87), and 0.53 (0.37–0.75), 0.3 (0.18–0.58), respectively (Figure **S3**). No significant heterogeneity was observed in the meta-analysis for these alleles. 

### *HLA-DPB1* alleles and PBC

A total of 13 meta-analyses have been conducted, but no statistically significant association between *HLA-DPB1* alleles and PBC risk was observed (data not shown).

### *HLA-DRB1* alleles and PBC

**Figure 3:**
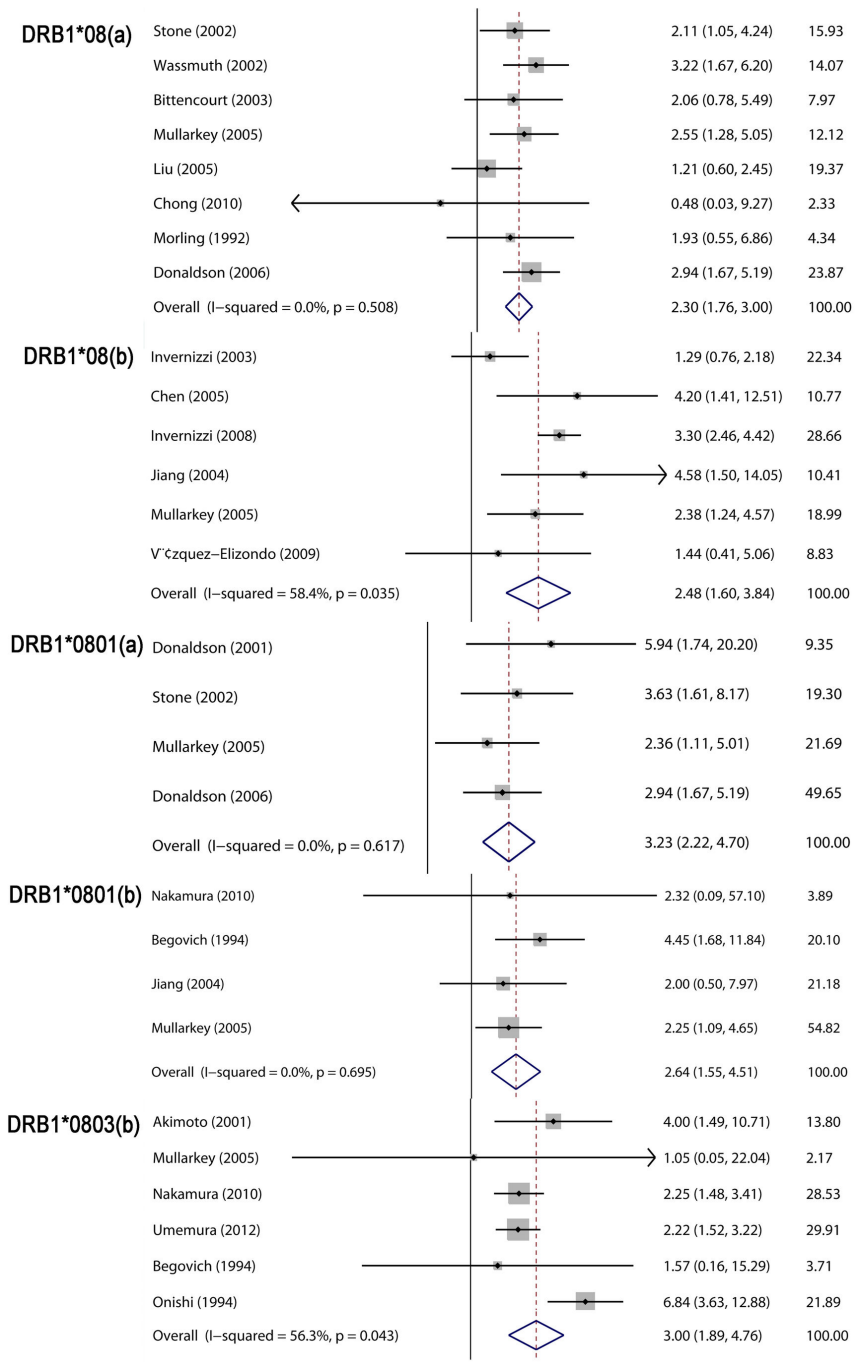
Meta-analysis of the studies of HLA-DRB1*08, *0801, *0803 and PBC risk. a: differences in the number of individuals who carried the *HLA-DRB1* allele, comparing PBC patients and controls. b: differences in the frequency of the *HLA-DRB1* allele between PBC patients and controls.

**Figure 4:**
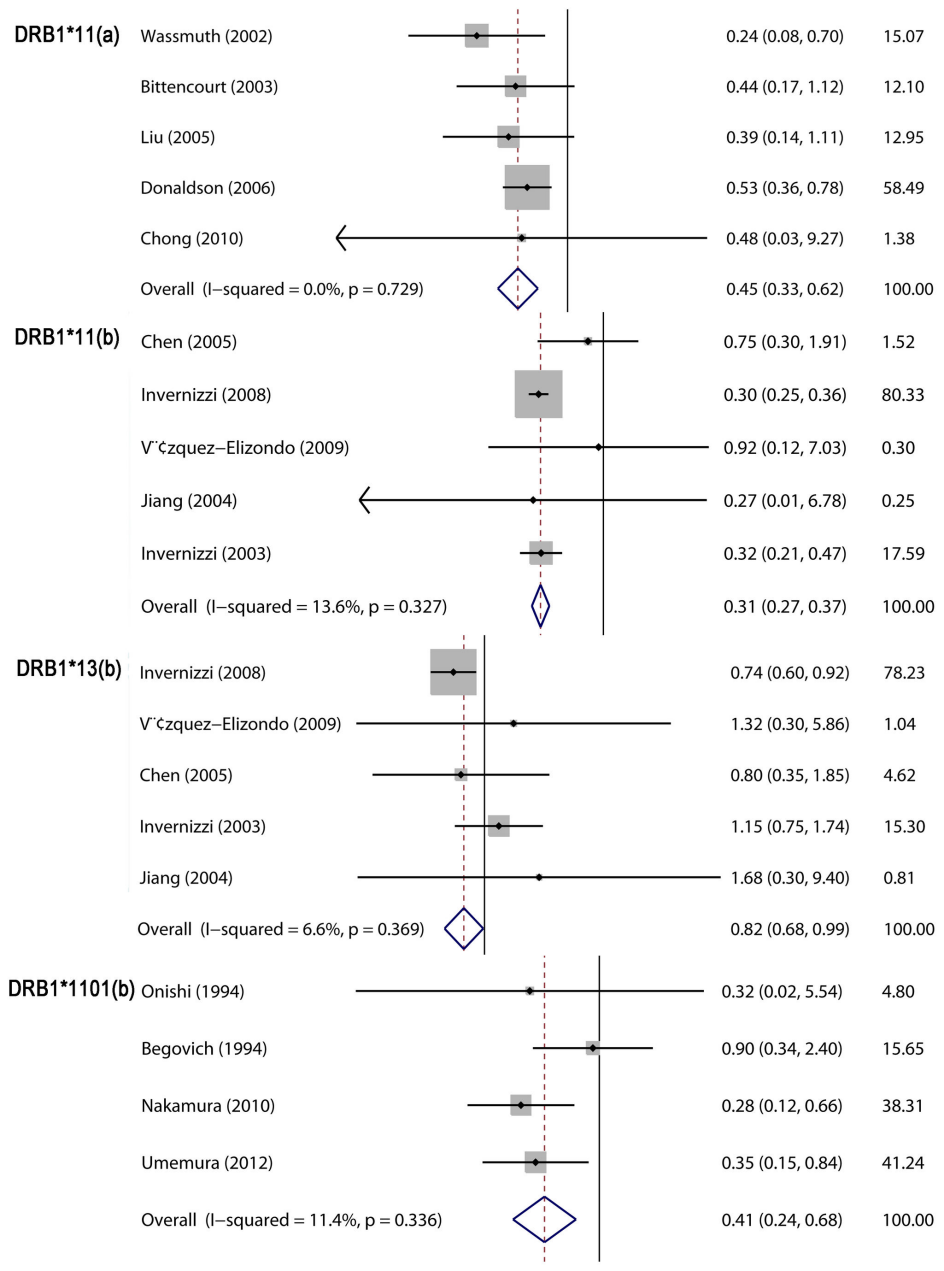
Meta-analysis of the studies of HLA-DRB1*11, *1101 and *13 and PBC risk.

Compared with *HLA-DQB1* and *HLA-DPB1* alleles, studies describing *HLA-DRB1* alleles and PBC were more abundant. *HLA-DRB1* alleles were also most extensively studied in the context of a relationship between *HLA-Class II* and PBC. *HLA-DRB1* (*01, *03, *0405, *07, *08, *0801 and *0803) were found to predisposition individuals to PBC, and the pooled results were 1.26 (1.05–1.51), 1.38 (1.16–1.65), 1.43 (1.16–1.76), 2.03 (1.29–3.21) and 1.47 (1.27–1.70), 2.30 (1.76–3.00) and 2.48 (1.60–3.84), 3.23 (2.22–4.70), and 2.64 (1.55–4.51), 3.00 (1.89–4.76), respectively (Figure **3**; Table **2**). Conversely, *HLA-DRB1* (*11, *13, *1101 and *1501) were observed to confer resistance to PBC, and the pooled results were 0.45 (0.33–0.62) and 0.31 (0.27–0.38), 0.41 (0.24–0.68), 0.82 (0.68–0.99), and 0.45 (0.31–0.65), respectively (Figure **4**; Table **2**). There was no significant heterogeneity in meta-analyses for most *HLA-DRB1* alleles, but high heterogeneity existed in meta-analyses for HLA-DRB1*08, *0803 and *13. The subgroup meta-analysis also demonstrated that HLA-DRB1*08 is a risk factor for PBC in Caucasian and Asian populations, but significant heterogeneity was observed in subgroup analyses. The subgroup meta-analysis for HLA-DR*0803 after stratification by ethnicity indicated that HLA-DR*0803 was a risk factor in Asian groups, not Caucasian groups. Also, the subgroup meta-analysis by ethnicity for HLA-DR*13 indicated that HLD-DR*13 was protective against PBC in Caucasian groups with a smaller heterogeneity. 

**Table 2:** Outcome, heterogeneity and publication bias tests for these meta-analyses.

	Number of		Heterogeneity			Publication	bias		
*HLA Class II*	studies included (N)	OR (95%CI)	Q test		I^2^ test	Begg' test		Egger' test	Association
*HLA-DR2*	7	0.70 (0.53–0.91)	0.372		7.4%	0.553		0.230	Protective factor
*HLA-DR5*	8	0.55 (0.39–0.78)	0.268		20.3%	0.902		0.610	Protective factor
*HLA-DR8*	12	2.82 (1.84–4.30)	0.011		54.8%	1		0.901	Risk factor
*HLA-DR12*	3	0.40 (0.19–0.86)	0.583		0	0.296		0.207	Protective factor
*HLA-DR16*	3	0.21 (0.05–0.97)	0.900		0	1		0.124	Protective factor
*HLA-DR52a*	3	0.60 (0.38–0.95)	0.459		0	1		0.613	Protective factor
HLA-DQB1*02 ^a^	3	1.40 (1.00–1.97)	0.204		37.2%	1		0.710	Risk factor
HLA-DQB1*0301 ^a^	3	0.62 (0.48–0.79)	0.468		0	1		0.997	Protective factor
HLA-DQB1*0301 ^b^	4	0.48 (0.35–0.68)	0.245		27.8%	1		0.558	Protective factor
HLA-DQB1*04 ^a^	4	2.24 (1.46–3.46)	0.692		0	0.308		0.247	Risk factor
HLA-DQB1*0401 ^b^	3	1.41 (1.07–1.85)	0.607		0	0.296		0.459	Risk factor
HLA-DQB1*0402 ^a^	4	3.47 (2.35–5.13)	0.560		0	0.308		0.150	Risk factor
HLA-DQB1*06 ^a^	3	0.58 (0.39–0.85)	0.433		0	1		0.836	Protective factor
HLA-DQB1*0601 ^b^	4	1.99 (1.57–2.53)	0.130		46.9%	1		0.778	Risk factor
HLA-DQB1*0602 ^a^	3	0.61 (0.42–0.87)	0.180		41.8%	0.296		0.229	Protective factor
HLA-DQB1*0602 ^b^	4	0.53 (0.37–0.75)	0.330		12.6%	1		0.589	Protective factor
HLA-DQB1*0604	3	0.3 (0.18–0.58)	0.667		0	0.296		0.328	Protective factor
HLA-DRB1*01 ^b^	6	1.26 (1.05–1.51)	0.134		40.7%	0.452		0.128	Risk factor
HLA-DRB1*03 ^*b*^	5	1.38 (1.16–1.65)	0.920		0	0.221		0.065	Risk factor
HLA-DRB1*0405 ^b^	4	1.43 (1.16–1.76)	0.749		0	0.734		0.328	Risk factor
HLA-DRB1*07 ^a^	3	2.03 (1.29–3.21)	0.415		0	1		0.874	Risk factor
HLA-DRB1*07 ^b^	5	1.47 (1.27–1.70)	0.836		0	0.806		0.083	Risk factor
HLA-DRB1*08 ^a^	8	2.30 (1.76–3.00)	0.508		0	0.035		0.185	Risk factor
HLA-DRB1*08 ^b^	6	2.48 (1.60–3.84)	0.035		58.4%	1		0.714	Risk factor
HLA-DRB1*0801 ^a^	4	3.23 (2.22–4.70)	0.617		0	0.308		0.272	Risk factor
HLA-DRB1*0801 ^b^	4	2.64 (1.55–4.51)	0.695		0	0.734		0.982	Risk factor
HLA-DRB1*0803 ^b^	6	3.00 (1.89–4.76)	0.043		56.3%	0.707		0.786	Risk factor
HLA-DRB1*11 ^a^	5	0.45 (0.33–0.62)	0.729		0	0.462		0.265	Protective factor
HLA-DRB1*11 ^b^	5	0.31 (0.27–0.38)	0.327		13.6%	0.806		0.190	Protective factor
HLA-DRB1*1101 ^*b*^	4	0.41 (0.24–0.68)	0.336		11.4%	0.734		0.965	Protective factor
HLA-DRB1*13 ^b^	5	0.82 (0.68–0.99)	0.369		6.6%	0.462		0.225	Protective factor
HLA-DRB1*1501 ^b^	3	0.45 (0.31–0.65)	0.186		40.6%	1		0.175	Protective factor

a indicated the difference in the number of individuals carried *HLA-DR*, *HLA-DQ*, *HLA-DP* allele between PBC patients and health controls.

b indicated the difference in the frequency of *HLA-DR*, *HLA-DQ* , *HLA-DP* allele between PBC patients and health controls.

### HLA haplotype and PBC

Of the 34 studies included, only 6 studies included discussions regarding the association between *HLA* haplotypes and PBC susceptibility [7,10,20,29,37,46]. Due to less overlap among *HLA* haplotypes detected in these 6 studies, a meta-analysis could not be conducted. 

### Sensitively analysis and assessment of bias

To assess the influence of each individual study on the overall OR, a sensitivity analysis was conducted by repeating the meta-analysis sequentially excluding one study at a time. Except for HLA-DRB1*13, the estimated pooled ORs were constant and the overall results were relatively stable in the present meta-analysis. The results indicate that the frequency of the HLA-DRB1*13 allele in PBC patients is significantly higher than in controls, but this relationship was not observed in individuals who carried the HLA-DRB1*13 allele between the PBC group and control group (OR = 0.668, 95%CI: 0.428–1.043; I^2^ = 59.0%, *P* = 0.045). However, after excluding Bittencourt’s study [39], the difference in the number of individuals who carried the HLA-DRB1*13 allele, comparing PBC patients and controls, achieved significance (OR = 0.553, 95%CI: 0.419–0.729), and this occurred with a lower heterogeneity (I^2^ = 13.8%, *P* = 0.323), supporting the hypothesis that inclusion of this particular study may lead to bias.

As shown in Table **2**, significant heterogeneity (*P* < 0.1) existed in the three meta-analyses including *DR8* and *HLA-DRB1* (*08, *0803). To investigate this finding, a meta-regression analyses for ethnicity, year of publication, and sample size was performed, and to avoid ecological bias and to limit type I errors, we perform no other exploratory regression analyses, especially for patient-level factors such as sex and age [48]. The meta-regression analysis showed that the major source of the heterogeneity listed in the meta-analyses for *HLA-DR8* and HLA-DRB1*08 could not be attributed to ethnicity, year of publication, or sample size (data not shown). However, the results obtained from meta-regression analysis for HLA-DRB1*0803 indicated that the year of publication may contribute to findings of heterogeneity, not ethnicity or sample size. After excluding two studies conducted before 2000 [7,9], significant heterogeneity disappeared (I^2^ = 0%, *P* = 0.677). The meta-analyses of HLA-DRB1*08 included a study in which PBC patients were a “mixed population”, which may explain heterogeneity. However, the heterogeneity in the meta-analysis of HLA-DRB1*08 did not decrease after excluding this study (I^2^ = 63.8%, *P* = 0.026) [42].

### Publication bias

The funnel plot for associations between *HLA Class II* and PBC susceptibility was drawn (data not shown) and publication bias was also measured by formal testing for funnel plot asymmetry using Egger’s test and Begg’s test. As expected, no significant publication bias was detected (Table **2**).

## Disscussion

To our knowledge, this is the first study to systemically review and meta-analyze all eligible published data to assess an association between *HLA Class II* and PBC risk. We discovered that common genetic variants of *HLA-Class II* were significantly associated with the development of PBC. A total of 26 *HLA Class II* alleles or antigens were found to be associated significantly with PBC as indicated by our meta-analysis of worldwide published data (Table **2**). Also, 13 of those variants were found to be significant risk factors for PBC including *DR8*, *HLA-DQB1* (*02, *04, *0401, *0402 and *0601), and *HLA-DRB1* (*01, *03, *07, *08, *0801, *0803 and *0405). Protective factors against PBC were found to include *HLA-DR2*, *DR5*, *DR12*, *DR16*, *DR52a*, *HLA-DQB1* (*06, *0301, *0602 and *0604) and *HLA-DRB1* (*11, *13, *1501, and *1101). No consistent conclusion regarding the association of *HLA* haplotypes and PBC risk could be drawn due to the small number of included studies. However, our findings provide important insight into the role of *HLA* class *II* in the immune-pathogenesis of PBC. 

A total of 34 studies were included in the present study (1983 to 2012), and 18 of these were determined to be of high quality; 16 were of medium quality; and no study was considered to be of low quality. Therefore, the meta-analysis presented here should reliably clarify which *HLA-Class II* genes may be responsible for PBC. 

Our data confirmed that HLA-DR*08, *0801, and *0803 were potential risk factors for PBC (corresponding serological type DR8). Previous studies indicated that HLA-DR8 was significantly associated with PBC, with ORs ranging from 2.4 to 3.3 based on the population examined [49]. In contrast, several reports have failed to confirm this association [30,33]. Nevertheless, evidence for this positive association was powerful in the present investigation, with the same direction of effect detected (OR = 2.82, 95%CI: 1.84–4.30). DRB1*08 was also observed to have a strong genetic association with PBC among many reports for Caucasian or Asian populations [8,10,38], but other studies failed to show a association between DRB1*08 and PBC [36,41,44]. 

Our work supports the hypothesis that both the number of individuals carrying the HLA-DRB1*08 allele and the frequency of the HLA-DRB1*08 allele in PBC groups were significantly greater than in control groups. Moreover, the results showed that HLA-DRB1*0801 and *0803 alleles conferred a predisposition to PBC. HLA-DRB1*08, *0801 and *0803 and HLA-DRB1*01, *03, *0405 and *07 were also identified to be risk-conferring alleles, but with relatively weak effects. Although the specific role of *HLA-Class II* risk alleles in PBC remains unclear, HLA molecules encoded by risk alleles such as *DR8*, might preferentially bind and present auto-antigens to T cells and trigger an autoimmune response [50,51]. Further studies are required to elucidate mechanisms by which *HLA-class II* genetic polymorphisms and pathogenesis cooperate in disease states such as PBC. 

Strong protective associations between HLA-DRB1*11, *1101, *13 and *1501 alleles and PBC were observed, and, although these findings have been replicated by several other studies, the data from these reports were inconsistent [39-41]. We therefore combined quantitative evidence from these studies to offer direct support for the conclusion that HLA-DRB1*11, *1101, *13 and *1501 alleles had strong protective effects against PBC, which more clearly integrates the relationship between *HLA-class II* and PBC [14]. Previous studies support the finding that HLA-DRB1*11 and *13 alleles were potent protective factors against some viruses [52-54]; thus, these *HLA-Class II* alleles may influence resistance to several infectious agents, and the lack of such alleles may lead to the onset of PBC through molecular mimicry of infectious agents. Several studies suggest that infection may be critical to the development of PBC [4,55,56], and our data also support a potential role for infections in PBC etiology.

Data derived from the GWAS of Canadian and Japanese patients indicated that *HLA-DQB1* had the strongest association with PBC. Here, we found 8 *HLA-DQB1* alleles that were associated with PBC and the association of the HLA-DQB1*0402 allele with PBC (OR 3.47, 95%CI 2.35–5.13) was strongest among them. All conclusions in 4 studies regarding the association between HLA-DQB1*0402 and PBC risk were consistent; this allele is a risk factor for PBC [10,20,31,38]. In contrast, the HLA-DQB1*03, *06, *0602, and *0604 alleles conferred protection against PBC. However, the meta-analyses indicated that no *HLA-DPB1* alleles were significantly associated with PBC. Most previous studies have also provided strong evidence for no association between *HLA-DPB1* and PBC. Until now, only two studies have suggested that HLA-DPB1*0301 and *0501 are associated with PBC, but these were based on relatively small sample populations [27,34]. A recent study from a large Italian cohort indicates that HLA-DPB1*0301 was a predisposing risk allele, a finding that is consistent with the previous study of a small German cohort [57]. However, as indicated in previous studies, our data support the finding that most *HLA* associations with PBC could be attributed to specific associations with *HLA-DRB1* and *HLA-DQB1* alleles rather than *HLA-DPB1*.

As mentioned before, the GWAS provided suggestive evidence for a strong association of PBC with *HLA* region polymorphisms. The strength of the association between *HLA-class* alleles and the risk of PBC in our current study was similar to data from other large-scale studies such as GWAS. In Juran’s study, 7 *HLA class II* alleles were reported to achieve genome-wide significance including HLA-DQA1*0501, *HLA-DQB1* (*0301, *0302 and *0402) and *HLA-DRB1* (*0801, *1101 and *1501) [58]. With the exception of HLA-DQB1*0302 and HLA-DQA1*0501, six other *HLA-class II* alleles were included in the present study. Data show that roles of these *HLA-class II* alleles in PBC development were quite consistent between Juran’s study and our study which suggested that HLA-DQB1*0402 and HLA-DRB1*0801 contribute to the PBC susceptibility and that conversely, HLA-DQB1*0301, HLA-DRB1*1101, and HLA-DRB1*1501 were protective alleles against PBC. Our estimated ORs were also quite similar to Juran’s results obtained from three independent large-scale cohorts. Collectively, this information indirectly reflects the accuracy and reliability of the present meta-analysis to study the role of *HLA* class *II* in PBC.

Studies to associate specific *HLA* haplotypes and susceptibility to PBC are scarce and those which exist indicate that several HLA haplotypes were significantly increased or reduced in PBC patients, suggesting that they confer susceptibility or protection to PBC, respectively. For example, the haplotype HLA-DRB1*0801-DQB1*0402 was identified as a risk factor for PBC in Caucasian subjects [7,34], and genes of the haplotype were found to be in linkage disequilibrium in the Caucasian population [46]. Furthermore, the frequency of the HLA-DRB1*0801-DQA1*0401-DQB1*0402 haplotype was reported to be increased in patients who had progressed to late stages of PBC, but not in those with early stage disease, suggesting that this haplotype may be a specific marker for the overall disease course [31]. We could not perform a meta-analysis to ascertain the association of *HLA* haplotype and PBC risk due to too few relevant original articles. Still, understanding and identifying these haplotype effects may improve research into or clinical treatment of PBC so future studies should be conducted to determine whether specific *HLA* haplotypes are associated with PBC susceptibility. 

Our meta-analysis had some limitations. First, we included a wide variety of articles to determine the role of *HLA* class *II* in PBC development, so specific differences within studies may be a potential source of bias. Second, all available studies were published data; unpublished data were not identified. This fact alone suggests that publication bias cannot be absolutely excluded even though no significant publication bias was observed by funnel plot analysis, Egger’ test, and Begg’s test in the meta-analyses. Next, it is also impossible to completely exclude the influence of confounding factors inherent in these included studies, although subgroup analyses by ethnicity were performed. Other confounders such as age, sex, country, specific technique could not be excluded, and this may explain our findings. Although we performed 32 meta-analyses, 11 of these only included 3 eligible studies. We could not conduct sufficient subgroup meta-analyses after stratification by relevant characteristics in each analysis. Finally, the data regarding PBC and other *HLA-Class II* alleles were extremely sparse and inadequate, limiting our ability to draw conclusions from the meta-analysis.

In summary, our investigations suggest that distinct *HLA* class *II* genetic variants conferred both a predisposition and a resistance to PBC. *HLA-DQB1* (*02, *04, *0401, *0402 and *0601) and *HLA-DRB1* (*01, *03, *0405, *07, *08, *0801, and *0803) were identified as risk factors for PBC, whereas *HLA-DQB1* (*0301, *06, *0602 and *0604), and *HLA-DRB1* (*11, *1101, *13 and *1501) were potent protective factors. Also, *DR8* was identified to be a predisposing factor. These results expand the repertoire of *HLA-Class II* genes with potential roles in PBC pathogenesis, however follow-up biological studies are needed to confirm these associations.

## Supporting Information

Checklist S1 PRISMA checklist.
(DOC)

Figure S1 Meta-analysis of the studies of HLA-DR serological antigens and PBC risk.
(TIF)

Figure S2 Meta-analysis of the studies of *HLA-DQ* risk alleles and PBC.
(TIF)

Figure S3 Meta-analysis of the studies of *HLA-DQ* protective alleles and PBC.
(TIF)

Table S1 Methodological quality of included studies according to the NEWCASTLE-OTTAWA Quality Assessment Scale.
(DOC)
