# Supplementary figures and images for: Association of Human Leukocyte Antigen Class II with Susceptibility to Primary Biliary Cirrhosis: A Systematic Review and Meta-Analysis
Source: PLoS One. 2013 Nov 12;8(11):e79580. doi: 10.1371/journal.pone.0079580 (PMC3827176; doi:10.1371/journal.pone.0079580)

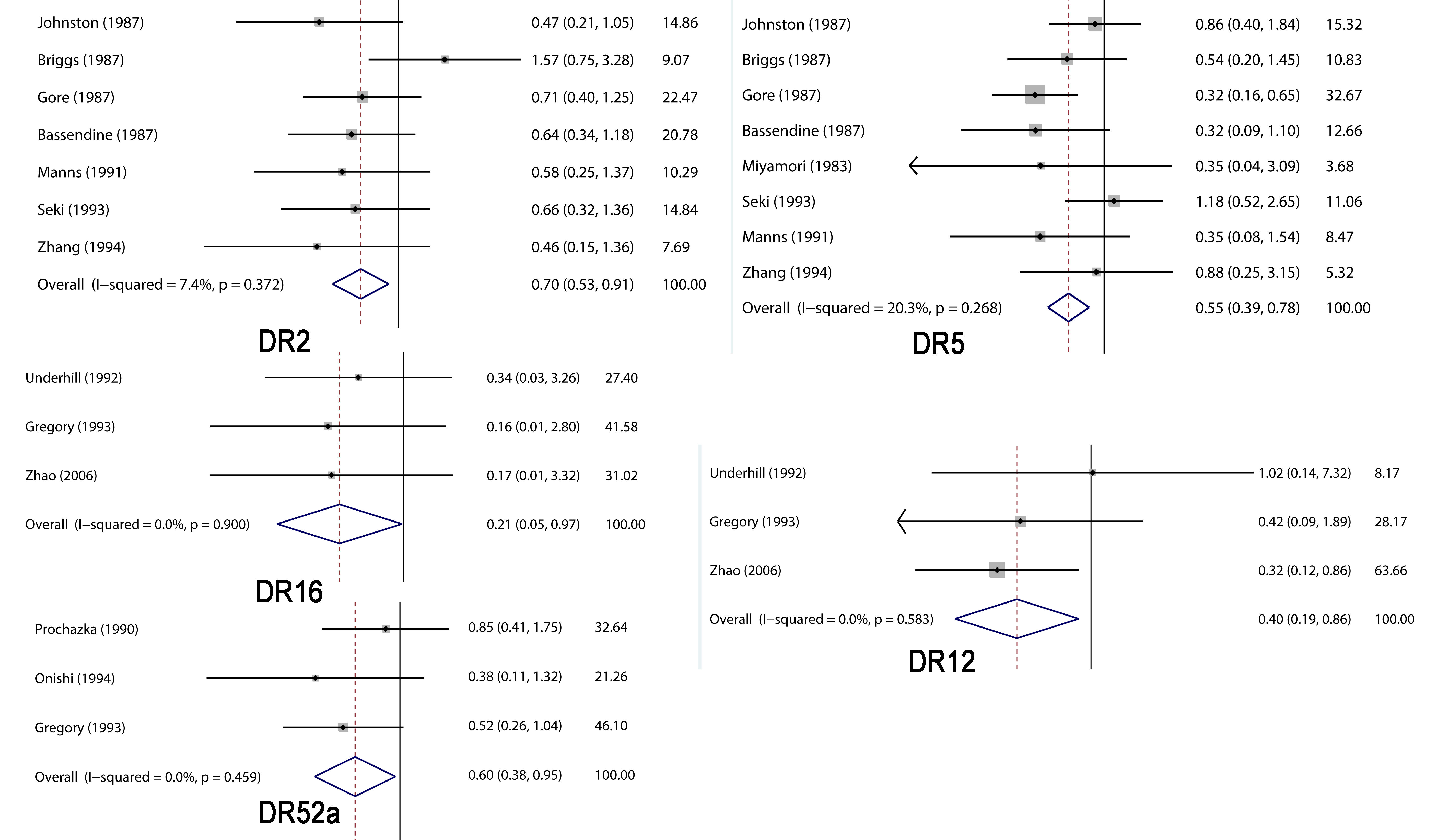

Supplement: Figure S1 — Meta-analysis of the studies of HLA-DR serological antigens and PBC risk. (TIF) [file pone.0079580.s002.tif]

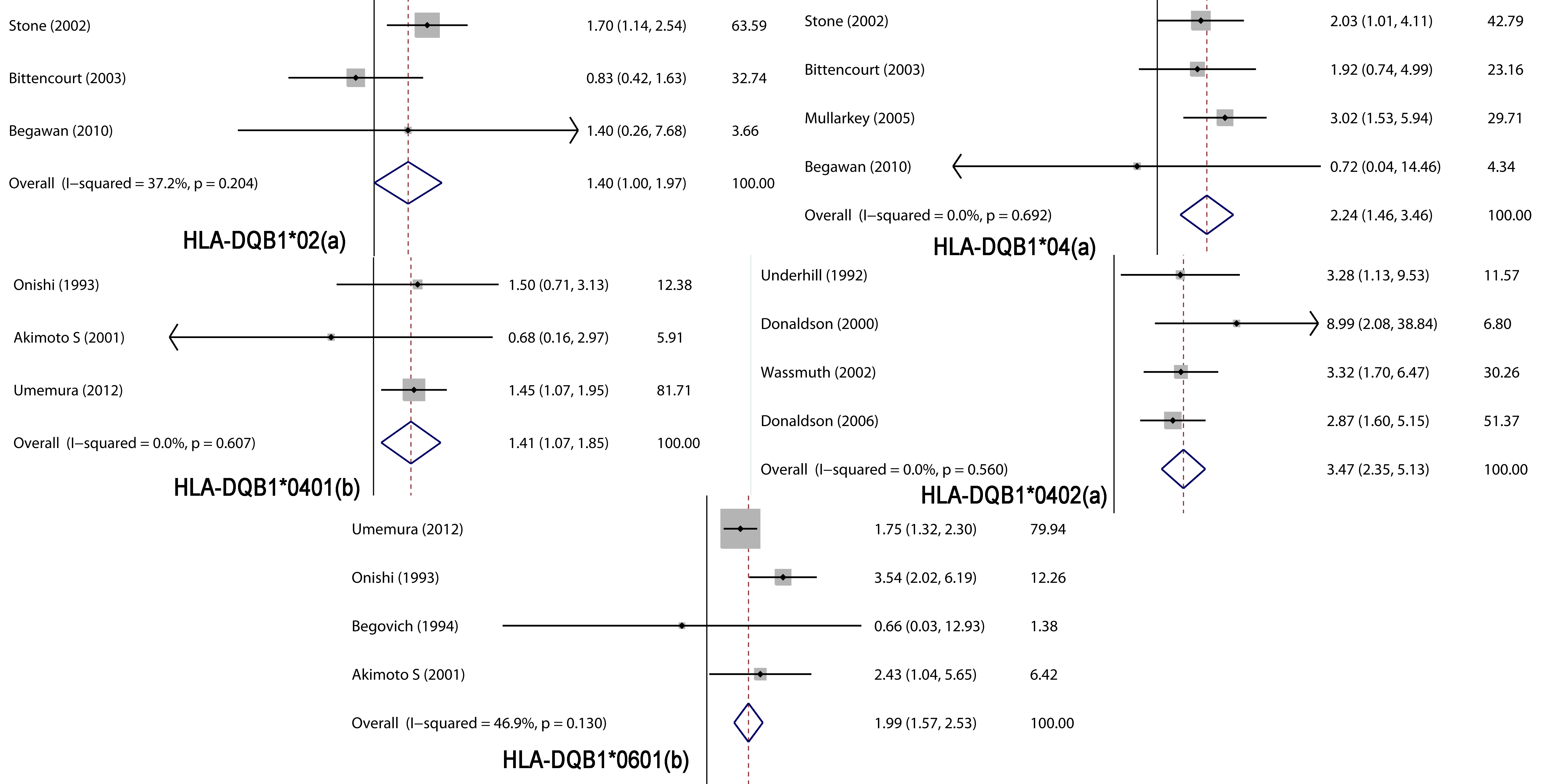

Supplement: Figure S2 — Meta-analysis of the studies of HLA-DQ risk alleles and PBC. (TIF) [file pone.0079580.s003.tif]

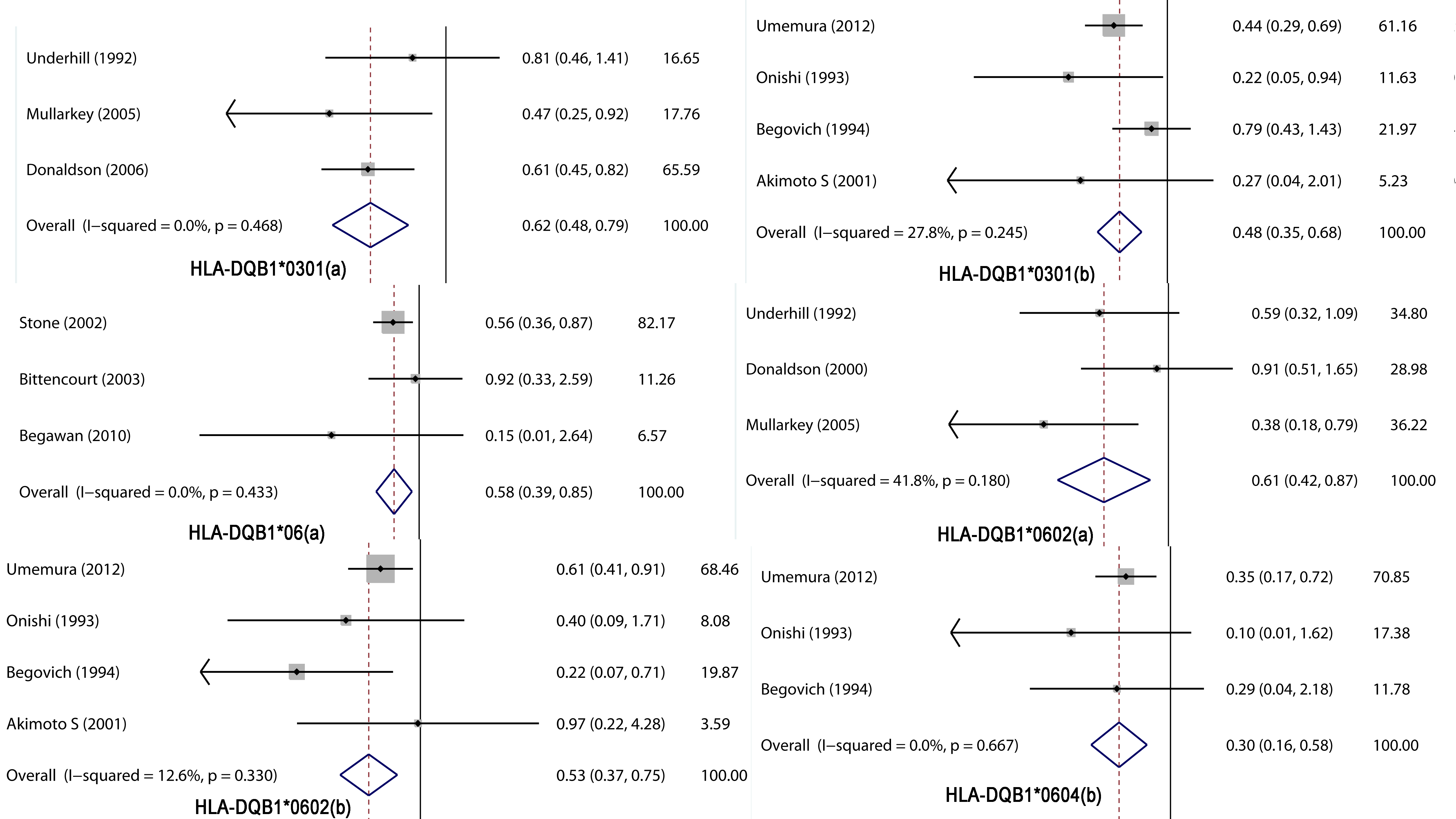

Supplement: Figure S3 — Meta-analysis of the studies of HLA-DQ protective alleles and PBC. (TIF) [file pone.0079580.s004.tif]
