# Supplementary material for: Association of Human Leukocyte Antigen Class II with Susceptibility to Primary Biliary Cirrhosis: A Systematic Review and Meta-Analysis
Source: PLoS One. 2013 Nov 12;8(11):e79580. doi: 10.1371/journal.pone.0079580 (PMC3827176; doi:10.1371/journal.pone.0079580)
Supplement: Table S1 — Methodological quality of included studies according to the NEWCASTLE-OTTAWA Quality Assessment Scale. (DOC) [file pone.0079580.s005.doc]

Sup.Table.1: Methodological quality of included studies according to the NEWCASTLE-OTTAWA Quality Assessment Scale

| **Study** | **Selection** | | | | **Comparability** | **Exposure** | | | **Total** |
| --- | --- | --- | --- | --- | --- | --- | --- | --- | --- |
|  | **Is the case definition adequate** | **Representativeness**  **of the cases** | **Selection of**  **controls** | **Definition of**  **controls** | **Comparability of cases and controls on the basis of the design or analysis** | **Ascertainment of exposure** | **Same method of ascertainment for cases and controls** | **Non-Response rate** |  |
| Underhill.JA,  et al | ★ | ★ | / | / | ★ | ★ | ★ | ★ | ★★★★  ★ |
| Morling N,  et al | ★ | / | / | / | ★ | ★ | ★ | / | ★★★★ |
| Manns MP,  et al | ★ | ★ | ★ | / | ★★ | ★ | ★ | / | ★★★★  ★★★ |
| Prochazka  EJ, et al | ★ | / | / | ★ | ★★ | ★ | ★ | ★ | ★★★★★★★ |
| Johnston DE, et al | ★ | ★ | / | / | ★ | ★ | ★ | ★ | ★★★★★★ |
| Briggs  DC, et al | ★ | ★ | ★ | / | ★★ | ★ | ★ | ★ | ★★★★★★★★ |
| Gores GJ,  et al | ★ | ★ | ★ | ★ | ★ | ★ | ★ | ★ | ★★★★★★★★ |
| Bassendine  MF, et al | ★ | / | ★ | ★ | ★ | ★ | ★ | / | ★★★★★★ |
| Miyamori H,  et al | ★ | / | ★ | ★ | ★ | ★ | ★ | / | ★★★★★★ |
| Onshi S.  et al | ★ | ★ | ★ | ★ | ★ | ★ | ★ | / | ★★★★★★★ |
| Underhill JA, et al | ★ | / | / | ★ | ★ | ★ | ★ | / | ★★★★★ |
| Mella JG,  et al | ★ | / | / | ★ | ★ | ★ | ★ | / | ★★★★★ |
| Zhang L,  et al | / | / | / | ★ | ★ | ★ | ★ | ★ | ★★★★★ |
| Gregory WL, et al | ★ | ★ | ★ | ★ | ★ | ★ | ★ | / | ★★★★★★★ |
| Mehal WZ, et al | ★ | ★ | ★ | ★ | ★ | ★ | ★ | ★ | ★★★★★★★★ |
| Begovich AB, et al | ★ | ★ | / | / | ★ | ★ | ★ | / | ★★★★★ |
| Seki T,  et al | ★ | ★ | ★ | ★ | ★★ | ★ | ★ | / | ★★★★★★★★ |
| Donaldson P, et al | ★ | ★ | ★ | / | ★★ | ★ | ★ | / | ★★★★★★★ |
| Akimoto S, et al | ★ | / | / | ★ | ★ | ★ | / | / | ★★★★ |
| Stone J,  et al | ★ | ★ | / | ★ | ★ | ★ | ★ | ★ | ★★★★★★★ |
| Wassmuth R, et al | ★ | ★ | / | ★ | ★ | ★ | ★ | ★ | ★★★★★★★ |
| Invernizzi P,  et al | ★ | ★ | / | ★ | ★ | ★ | ★ | / | ★★★★★★ |
| Bittencourt PL, et al | ★ | ★ | ★ | ★ | ★★ | ★ | ★ | / | ★★★★★★★★ |
| Jiang XH,  et al | ★ | / | ★ | ★ | ★ | ★ | ★ | / | ★★★★★★ |
| Mullarkey ME, et al | ★ | ★ | / | ★ | ★ | ★ | ★ | ★ | ★★★★★★★ |
| Liu HY,  et al | ★ | ★ | ★ | ★ | ★ | ★ | ★ | / | ★★★★★★★ |
| Donaldson PT, et al | ★ | ★ | / | ★ | ★ | ★ | ★ | ★ | ★★★★★★★ |
| Invernizzi P, et al | ★ | ★ | ★ | ★ | ★ | ★ | ★ | ★ | ★★★★★★★★ |
| Vázquez-  Elizondo  G, et al | ★ | ★ | ★ | ★ | ★ | ★ | ★ | ★ | ★★★★★★★★ |
| Nakamura M, et al | ★ | ★ | / | / | ★ | ★ | ★ | / | ★★★★★ |
| Chong VH, et al | ★ | ★ | / | / | ★ | ★ | ★ | / | ★★★★★ |
| Umemura T, et al | ★ | ★ | / | ★ | ★ | ★ | ★ | / | ★★★★★★ |
| Chen C,  et al | ★ | ★ | / | / | ★ | ★ | ★ | ★ | ★★★★★★ |
| Zhao J,  et al | ★ | ★ | ★ | ★ | ★ | ★ | ★ | / | ★★★★★★★ |
